# Supplementary material for: German Language Adaptation of the NAVS (NAVS-G) and of the NAT (NAT-G): Testing Grammar in Aphasia
Source: Brain Sci. 2021 Apr 8;11(4):474. doi: 10.3390/brainsci11040474 (PMC8069474; doi:10.3390/brainsci11040474)
Supplement: Supplementary file 1 [file brainsci-11-00474-s001.pdf]

Supplementary Materials and Results: The following are available online at www.mdpi.com/xxx/s1

Contents:

Supplementary Materials

- Supplementary Table 1: Summary of tests for aphasia in German language
- Supplementary Table 2: The healthy participants
- Supplementary Table 3: The aphasic participants
- Supplementary Table 4: List of changed target verbs in NAVS-G /NAT-G compared to original NAVS-G
- Supplementary Table 5: List of log. frequency of each target verb used in NAVS-G /NAT-G
- Supplementary Table 6: List of novel sentences in NAVS-G /NAT-G
- Supplementary Figure 1: NAVS-G errors in patients with aphasia

Supplementary results: Within group analysis

- Supplementary Table 7: All participants´ results in the different subtests
- Supplementary Table 8: Complete NAVS-G VNT results
- Supplementary Table 9: Complete NAVS-G ASPT results
- Supplementary Table 10: Complete NAVS-G SPPT results
- Supplementary Table 11: Complete NAVS-G SCT results
- Supplementary Table 12: Complete NAT-G results

Supplementary Materials

Supplementary Table1 1: Overview of aphasia tests in the German language

German Tests for Aphasia	Comprehension		Production				Reading
	Sentences	Syntax	Repetition	Naming	Sentence	syntax	
Aachener Aphasie Test	x		x	x	x	(x)	x
Aachener Aphasie Bedside-Test	x			x	x		
Aphasie-Check-Liste	x		x	x			x
Amsterdam-Nijmegen Everyday Language Test							
Aphasie-Schnell-Test	x						x
Bielefelder Aphasie Screening	x		x	x	x		x
Kurze Aphasieprüfung	x		x	x			x
Komplexe Sätze					x	x	
Sätze verstehen	x	x					
Lexikon modellorientiert 2.0	x		x	x			x
Sprachsystematisches Aphasiescreening	x	x	x	x	x	x	
Token-Test	x					(x)	
NAVS	x	x		x	x	x	
NAT					x*		(x)

Supplementary Table 1 gives an overview of frequently used German language tests. It compares three aspects of speech: “comprehension” (subdivided in “sentence comprehension” and “syntax”). “production” (subdivided in “repeating”, “naming”, “sentence production”, and “syntax”), and “reading”.

The Bielefelder Aphasie Screening (Richter et al. 2006) is another diagnostic test for German stroke patients, allowing aphasia's diagnosis, aphasia's severity, and the syndrome classification. Basic syntactic skills are examined in several subtests (the analysis of spontaneous speech, sentence comprehension, and sentence production for picture description). However, a depth-diagnostics concerning syntax, e.g., the production of complex sentences, is missed. In contrast to the above-mentioned traditional aphasia test batteries, the "Lexikon modellorientiert 20" (LeMO) is based on the language faculty's cognitive neuropsychological models (Morton 1979 and 1970) to aphasia assessment and to plan individual treatment. This model lacks the syntax system. Therefore, the LEMO could not identify syntactic deficits.

Characteristics of Healthy and aphasic participants

Supplementary Table 2: The healthy participants

Characteristics of Participants				
**Subject**	**Gender**	**Age in years**	**Educational qualification**	**Highest educational degree**
1	f	60	3	3
2	m	60	3	2
3	f	64	3	2
4	f	49	3	2
5	m	57	4	3
6	m	48	4	3
7	f	57	4	2
8	f	62	4	3
9	m	66	4	3
10	f	28	4	3
11	m	30	4	3
12	m	25	4	3
13	f	25	4	3
14	f	29	4	3
15	m	34	4	3
16	m	34	4	3
17	m	52	2	2
18	f	79	2	1
19	f	68	3	1
20	m	78	3	2
21	f	72	3	2
22	m	77	2	2
23	m	75	2	2
24	m	52	4	3
25	f	84	1	1
6	f	88	1	1
27	m	56	2	1

Supplementary Table 2 reports: gender - f= female. m= male - education (Ed.) qualification - 1 = No school leaving certificate. 2 = junior high school certificate covering eight years (“Hauptschulabschluss”). 3 = secondary high school leaving certificate covering ten years (“Realschulabschluss”). 4 = Abitur -, and highest educational qualification - 1= no education degree. 2= completed apprenticeship degree. 3 = university degree.

Pat.	*infarct size*	*Aphasia (T0)*			*Aphasia (T1)*									*MoCA(T1)*								*NAVS-G /NAT-G*
		**Severity**	**Type**	**GS**	**severity**	**Type**	**GS**	**SS**	**TT**	**R**	**W**	**N**	**C**	**GS**	Ex	N	At	L	Ab	M	O	**GS**
	ml			0-530			0-530	0-30	0-50	0-150	0-90	0-120	0-120	0-30	0-5	0-3	0-6	0-3	0-2	0-5	0-6	%
**1**	47.24	middle	Global	213	mild	Broca	457	25	44	135	75	105	98									76
**2**	79.59	middle	Broca	237	mild	Anomic	471	25	48	136	77	103	94	18	2	3	4	1	1	2	5	90
**3**	38.64	severe	Global	182	mild	Anomic	446	26	40	139	83	83	101									98
**4**	7.26	mild	Anomic	447	minimal	RA	#	28	50	^	89	^	^	24	4	3	6	2	1	2	6	95
**5**	1.81	mild	Broca	460	minimal	RA	#	29	50	^	89	^	^	26	5	3	6	2	2	2	6	98
**6**	24.96	minimal	RA	512	minimal	RA	#	29	50	^		^	^	25	4	2	6	1	2	4	6	98
**7**	35	mild	Anomic	416	minimal	RA	#	29		^		^	^	27	4	3	6	2	1	4	6	97
**8**	106.99	severe	Global	64	minimal	RA	516	27	45	148	89	120	114									98
**9**	18.48	very severe	Global	#	minimal	RA	490	21	49	130	83	114	114									95
**10**	3.23	very severe	Global	#	mild	Broca	394	19	27	124	51	81	70	#								81
**11**	16.18	very severe	Global	#	middle	Broca	378	16	47	94	51	82	59	#								67
**12**	^	mild	Anomic	#	minimal	RA	#	28	46	^	90	^	^	25	5	1	6	2	2	3	6	82
**13**	*	severe	Broca	#	mild	Broca	*	21	*	*	*	*	*	*	*	*	*	*	*	*	*	92
**14**	*	mild	Anomic	*	minimal	RA	*	29	*	*	*	*	*	*	*	*	*	*	*	*	*	98
**15**	34.5	mild	Anomic	476	minimal	RA	513	29	45	150	90	119	109	28	5	3	6	3	2	3	6	76

Supplementary Table2: The aphasic participants

Supplementary Table 3 reports the AAT results of the patients with aphasia at stroke time and NAVS-G and NAT-G testing (chronic time). Patients' row data of the AAT's six description levels for spontaneous speech (including communicative verbal behavior (1), articulation and prosody (2), automatized language (3), semantic structure (4), phonemic structure (5), and syntactic structure (6)), and the row data of the five AAT's subtests (token test (TT), repetition (R), written language (WR), naming (N), and sentence comprehension (C)) are reported for the chronic time. A total of 530 points can be achieved in the AAT. Patients N. 13 and 14 had a clinical diagnosis of aphasia based on AAT spontaneous speech analysis only, as AAT's testing was not available. Patient n. 6 refused further testing.

MoCA test could be performed only in patients who participated in Large scale protocol and when the clinic profile was light. The character # dispays patients who could perform neither the AAT at the acute phase nor the MoCA in the chronic phase. The MOCA includes seven subtests for testing executive function (Ex), naming (N), attention (A), language (L), abstraction (Ab), verbal memory (M), and orientation (Or). In bracket are noticed the maximal scores for each subtest.

The table also reports the NAVS/NAT-G global scores (GS) for each patient.

Abbreviation: RA = residual aphasia

List of novel verbs in NAVS-G /NAT-G

Supplementary Table 3: List of changed target verbs in NAVS-G /NAT-G compared to original NAVS-G

Verb in NAVS (original version)	German translation	alternative Verb in NAVS-G /NAT-G	Reason for change
cut (ob2)	schneiden (ob2)	küssen (ob2)<br>transl.: *kiss*	NAT verb
read (op3)	**vor**lesen (op3)	schreiben (op3)<br>transl.: *write*	exclusion of compound verbs
watch (op2)	beobachten (ob2)	essen (op2)<br>transl.: *eat*	different argrument structure
stir (ob2)	rühren (op2)	retten (ob2)<br>transl.: *save*	different argrument structure
pinch (ob2)	kneifen (ob2)	jagen (ob2)<br>transl.: *chase*	NAT verb
pour (op3)	**ein**schenken (op3)	zeigen (op3)<br>transl.: *show*	exclusion of compound verbs
tickle (ob2)	kitzeln (ob2)	ziehen (ob2)<br>transl.: *pull*	NAT verb
shave (op2)	rasieren (ob2)	fegen (op2)<br>transl.: *sweep*	different argrument structure

We substituted: 1) 'to watch' (beobachten) and 'to shave' (rasieren), as they are op2 in Englisch but ob2 in German. To stir' (rühren) is ob2 in English but op2 in German; 2) 'to pour' (einschenken) and 'to read' (vorlesen). as their translation to German is a compound verb; 3) 'to cut'. 'to pinch' and 'to tickle' were replaced by 'to kiss' - küssen-. 'to chase' - jagen- and 'to pull' - ziehen- other ob2-verbs used in the sentences in the original NAT.

Frequency of verbs used in NAVS-G and NAT-G.

Supplementary Table 4: List of log. frequency of each target verb used in NAVS-G /NAT-G

Verb	Argument structure	log. frequency
bellen (bark)	ob1	.4771
heulen (howl)	ob1	.8451
krabbeln (crawl)	ob1	0
lachen (laugh)	ob1	20374

schwimmen (swim)	ob1	1.415
**ob1 mean**		**.9549**
jagen (chase)	ob2	1.4472
küssen (kiss)	ob2	1.4914
retten (save)	ob2	1.8573
schubsen (shove)	ob2	0
ziehen (pull)	ob2	2.4533
**ob2 mean**		**1.4498**
fahren (drive)	op2	2.6857
waschen (wash)	op2	1.301
beißen (bite)	op2	1.3222
essen (eat)	op2	1.8261
fegen (sweep)	op2	.7782
**op2 mean**		**1.5827**
stellen (put)	ob3	2.8457
geben (give)	ob3	3.2378
**ob3 mean**		**3.0418**
schicken (send)	op3	1.7634
schreiben (write)	op3	2.4857
überreichen (deliver)	op3	1.5051
werfen (throw)	op3	2.1818
zeigen (show)	op3	2.7226
**op3 mean**		**2.1317**

List of novel sentences in NAVS-G /NAT-G

Supplementary Table 5: List of novel sentences in NAVS-G /NAT-G

Test	German Verb (English translation)	novel sentence in ASPT
ASPT	küssen (kiss)	Der Mann küsst die Frau.
	schreiben (write)	Der Mann schreibt (der Frau) einen Brief.

	essen (eat)	Der Mann isst (ein Brötchen).
	retten (save)	Der Mann rettet die Frau.
	jagen (chase)	Der Hund jagt die Katze.
	ziehen (pull)	Der Junge zieht das Mädchen.
	fegen (sweep)	Der Mann fegt (den Boden).
NAT/SPPT/SVT	schubsen (shove)	z.B. Aktiv: Der Junge schubst das Mädchen

MRI data acquisition and lesion analysis

For the diffusion-weighted imaging. we used a standard sequence (23 slices. matrix = 128 x128 pixel. voxel size = 1.8 x1.8 x 5 mm. repetition time = 3.1 s. echo time = 79 ms. flip angle = 9. six diffusion-encoding gradient directions with a b-factor of 1000 s/mm2). All patients received FLAIR images (repetition time = 9000 ms. echo time = 93.0 ms. flip angle = 14. matrix 200 x 256 pixel. voxel size = 0.94 x 0.94 x 5.00 mm. 23 slices). As a prerequisite for spatial normalization. a high-resolution T1 anatomical scan was obtained from 91 patients (repetition time = 2200 ms. echto time = 2.15 ms. flip angle = 12. matrix = 256 x 256 pixel. voxel size = 1 x 1 x 1 mm. 176 slices).

For lesion analysis. a rough delineation of the diffusion-weighted imaged lesion was performed using a customized region-of-interest toolbox implemented in SPM8 (http://www.fil.ion.ucl.ac.uk/spm/software/spm8). Individual intensity thresholds were applied to find the best match between the binary lesion map and the diffusion-restricted brain tissue. Subsequently. the lesions maps were inspected in MRIcron (http://www.cabiatl.com/mrico/mricon/stats.html) and manually adjusted if necessary. In one case. no diffusion-weighted image was available and the lesion was drawn directly onto a FLAIR image. For spatial normalization of the lesion maps. the underlying diffusion-weighted imaging scan (or FLAIR image) was co-registered to the anatomical T1 scan (n = 91). High-resolution T1 scans were segmented using the VBM8 toolbox (r435; http://dbm.neuro.uni-jena.de/vbm/ download/). Deformation field parameters for nonlinear normalization into the stereotactic Montreal Neurological Institute (MNI) standard space were then computed using the DARTEL (diffeomorphic anatomical registration through exponentiated lie algebra; [177]) approach implemented in VBM8. Normalization quality of lesion maps was visually checked by M.H. In five cases in which no T1 scan of sufficient quality was available. parameters for normalization were obtained using FLAIR images. As smaller diffusion restricted areas may be indiscernible in chronic MRI scans or CT scans. our approach of mapping lesions on acute diffusion-weighted images may contribute significantly to a more precise determination of lesion.

NAVS-G and NAT-G errors

- **In the control groups**

Healthy participants and right hemispheric stroke patients named all verbs correctly. Nevertheless. they used synonyms

I. frieren statt *zittern* (freeze instead of *shiver*)

II. ablegen/hinlegen/hinstellen instead of *stellen* (put down/put away instead of *put*

III. parken instead of *fahren* (park instead of *drive*)

IV. herausziehen/herausholen instead of *retten (*pull out instead of *rescue*)

V. schenken, instead of *geben (*donate instead of *give)*

VI. kraulen, instead of *schwimmen* (swim the crawl instead of swim)

VII. verfolgen. nachgehen (Dialect), instead of *jagen (*to pursue (dialect) instead of to hunt)

VIII. kriechen, instead of *krabbeln (*crawl instead of crawling)

IX. jaulen, instead of *heulen*

X. kehren oder schweifen (Dialekt) statt fegen (sweep or ramble (dialect) instead of sweep)

- **in stroke patients with aphasia**

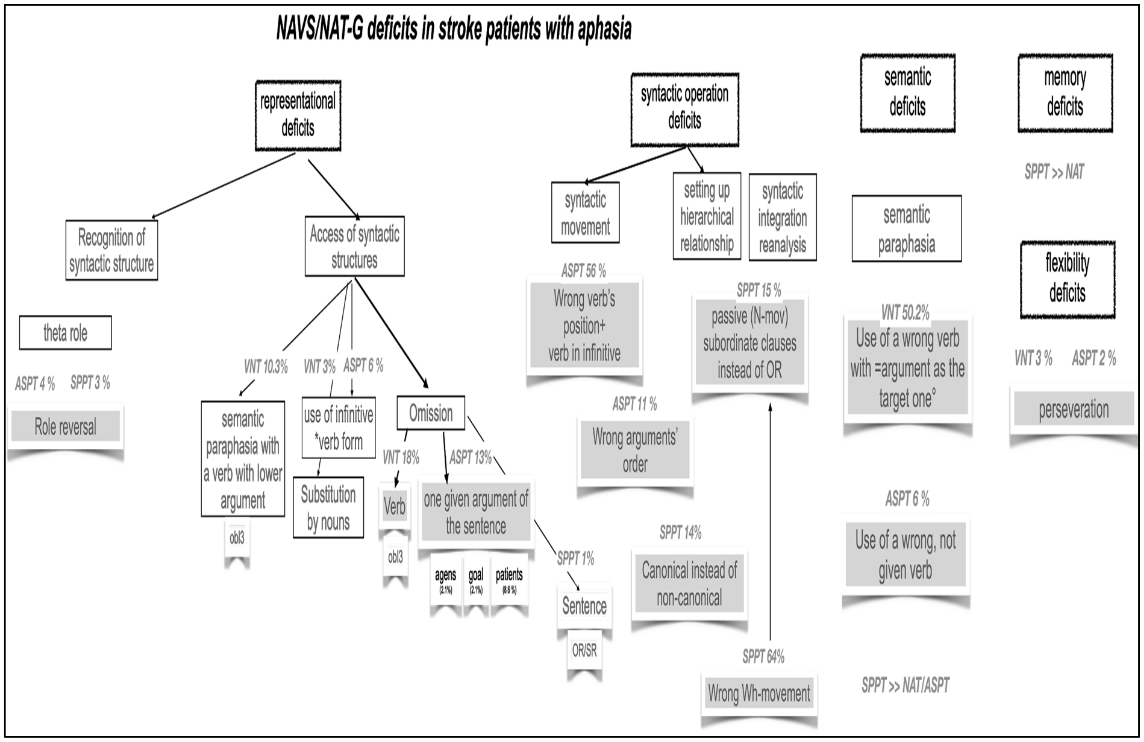

Supplementary Figure 2: NAVS-G errors in patients with aphasia

Patients with aphasia produced several kinds of mistakes when performing the NAVS-G and NAT-G tests. According to the literature, differentiating between representational deficits, deficits in syntactic operation, and semantic errors is, however, possible.

Supplementary results: Descriptive statistics

Supplementary Table 6: Participants´ results in the different NAVS-G and NAT-G subtests

N.	VNT	VCT	ASPT	NAT	NAT can	NAT non-can	NAT OR	SPTT	SPPT can.	SPPT non-can.	SPPT OR	SVT total	SVT can.	SVT non-can.	total score
LHSP1	81.8%	100%	**84.7%**	**53.3**%	60%	46.7%	40%	3.0%	46.7%	13.3%	.0%	80%	100%	60%	76.3%
LHSP 2	95.5%	100%	100%	**83.3%**	100%	66.7%	20%	40%	46.7%	33.3%	20%	83.3%	86.7%	80%	90%
LHSP 3	100%	100%	100%	**96.7%**	100%	93.3%	100%	86.7%	93.3%	80%	40%	100%	100%	100%	98.3%
LHSP 4	86.4%	100%	**96.8%**	100%	100%	100%	100%	86.7%	93.3%	80%	40%	90%	100%	80%	94.8%
LHSP 5	95.5%	100%	100%	100%	100%	100%	100%	90%	93.3%	86.7%	60%	96.7%	100%	93.3%	98.3%
LHSP 6	100%	100%	100%	100%	100%	100%	100%	80%	86.7%	73.3%	20%	100%	100%	100%	97.9%
LHSP 7	86.4%	100%	100%	90%	93.3%	86.7%	60%	90%	93.3%	86.7%	60%	96.7%	100%	93.3%	96.6%
LHSP 8	100%	100%	**99.4%**	96.7%	100%	93.3%	80%	86.7%	93.3%	80%	40%	100%	100%	100%	97.9%
LHSP 9	99%	100%	100%	96.7%	100%	93.3%	80%	66.7%	100%	33.3%	.0%	96.7%	100%	93.3%	95.2%
LHSP 10	86.4%	100%	100%	20%	33.3%	6.7%	.0%	23.3%	40%	67%	.0%	80%	93.3%	66.7%	8.8%
LHSP 11	72.7%	95.5%	**71%**	46.7%	66.7%	26.7%	.0%	36.7%	53.3%	20%	.0%	76.7%	93.3%	60%	67.0%
LHSP 12	86.4%	100%	**96.2**%	33.3%	60%	6.7%	.0%	40%	80%	.0%	.0%	83.3%	86.7%	80%	82.1%
LHSP 13	99%	100%	100%					53.3%	60%	46.7%	.0%	83.3%	66.7%	100%	82.5%
LHSP 14	99%	100%	100%					90%	93.3%	86.7%	60%	96.7%	100%	93.3%	87.6%
LHSP 15	77.3%	100%	100%	100%	100%	100%	100%	80%	100%	60%	40%	100%	100%	100%	76.3%
HP1	100%	100%	1000%	100%	100%	100%	100%	100%	100%	100%	100%	100%	100%	100%	100%
HP2	100%	100%	1000%	100%	100%	100%	100%	96.7%	100%	93.3%	80%	100%	100%	100%	99.7%
HP3	100%	100%	1000%	100%	100%	100%	100%	100%	100%	100%	100%	100%	100%	100%	100%
HP4	100%	100%	1000%	100%	100%	100%	100%	100%	100%	100%	100%	100%	100%	100%	100%
HP5	100%	100%	1000%	100%	100%	100%	100%	96.7%	100%	93.3%	80%	100%	100%	100%	99.7%

HP6	100%	100%	1000%	100%	100%	100%	100%	100%	100%	100%	100%	100%	100%	100%	100%
HP7	100%	100%	1000%	100%	100%	100%	100%	100%	100%	100%	100%	100%	100%	100%	100%
HP8	100%	100%	1000%	100%	100%	100%	100%	100%	100%	100%	100%	100%	100%	100%	100%
HP9	100%	100%	1000%	100%	100%	100%	100%	96.7%	100%	93.3%	100%	100%	100%	100%	99.7%
HP10	100%	100%	1000%	100%	100%	100%	100%	100%	100%	100%	100%	100%	100%	100%	100%
HP11	100%	100%	1000%	100%	100%	100%	100%	100%	100%	100%	100%	100%	100%	100%	100%
HP12	100%	100%	1000%	100%	100%	100%	100%	100%	100%	100%	100%	100%	100%	100%	100%
HP13	100%	100%	1000%	100%	100%	100%	100%	100%	100%	100%	100%	100%	100%	100%	100%
HP14	100%	100%	1000%	100%	100%	100%	100%	100%	100%	100%	100%	100%	100%	100%	100%
HP15	100%	100%	1000%	100%	100%	100%	100%	100%	100%	100%	100%	100%	100%	100%	100%
HP16	100%	100%	1000%	100%	100%	100%	100%	100%	100%	100%	100%	100%	100%	100%	100%
HP17	100%	100%	1000%	100%	100%	100%	100%	76.7%	93.3%	60%	.0%	100%	100%	100%	97.6%
HP18	95.5%	100%	1000%	100%	100%	100%	100%	93.3%	93.3%	93.3%	80%	100%	100%	100%	990%
HP19	100%	100%	1000%	100%	100%	100%	100%	96.7%	100%	93.3%	80%	100%	100%	100%	99.7%
HP20	100%	95.5%	1000%	100%	100%	100%	100%	93.3%	100%	86.7%	60%	100%	100%	100%	990%
HP21	100%	100%	1000%	100%	100%	100%	100%	100%	100%	100%	100%	100%	100%	100%	100%
HP22	95.5%	100%	1000%	100%	100%	100%	100%	86.7%	86.7%	86.7%	60%	86.7%	100%	73.3%	96.9%
HP23	100%	100%	1000%	100%	100%	100%	100%	86.7%	100%	73.3%	20%	96.7%	93.3%	100%	98.3%
HP24	100%	100%	1000%	100%	100%	100%	100%	100%	100%	100%	100%	100%	100%	100%	100%
HP25	100%	100%	1000%	100%	100%	100%	100%	83.3%	100%	66.7%	.0%	100%	100%	100%	98.3%
HP26	100%	100%	1000%	100%	100%	100%	100%	76.7%	93.3%	60%	20%	100%	100%	100%	97.6%
HP27	100%	100%	1000%	100%	100%	100%	100%	100%	100%	100%	100%	100%	100%	100%	100%
RHSP1	100%	100%	100%	100%	100%	100%	100%	100%	100%	100%	100%	100%	100%	100%	100%
RHSP2	100%	100%	100%	100%	100%	100%	100%	80%	86.7%	73.3%	40%	100%	100%	100%	97.9%
RHSP3	100%	100%	100%	93.3%	100%	86.7%	60%	80%	93.3%	66.7%	40%	100%	100%	100%	97.3%
RHSP4	100%	100%	100%	100%	100%	100%	100%	90%	93.3%	86.7%	80%	100%	100%	100%	990%
RHSP5	100%	100%	100%	100%	100%	100%	100%	96.7%	100%	93.3%	80%	100%	100%	100%	99.7%
RHSP6	100%	100%	100%	100%	100%	100%	100%	93.3%	100%	86.7%	80%	100%	100%	100%	99.3%
RHSP7	100%	100%	100%	86.7%	93.3%	80%	40%	86.7%	86.7%	86.7%	60%	90%	93.3%	86.7%	96.2%
RHSP8	100%	100%	100%	100%	100%	100%	100%	100%	100%	100%	100%	100%	100%	100%	100%
RHSP9	100%	100%	100%	100%	100%	100%	100%	100%	100%	100%	100%	100%	100%	100%	100%
RHSP10	100%	100%	100%	100%	100%	100%	100%	100%	100%	100%	100%	100%	100%	100%	100%
RHSP11	95.5%	100%	100%	100%	100%	100%	100%	86.7%	100%	73.3%	60%	93.3%	100%	86.7%	97.6%

RHSP12	100%	100%	100%	100%	100%	100%	100%	96.7%	100%	93.3%	80%	100%	100%	100%	99.7%
RHSP13	100%	100%	100%	100%	100%	100%	100%	93.3%	100%	86.7%	80%	100%	100%	100%	99.3%
RHSP14	100%	100%	100%	100%	100%	100%	100%	100%	100%	100%	100%	100%	100%	100%	100%
RHSP15	100%	100%	100%	93.3%	100%	86.7%	60%	90%	100%	80%	60%	100%	100%	100%	98.3%

Complete results of the logistic regression

Please note that the effect is positive or negative depending on whether the z/X^2 score is positive or negative respectively

Supplementary Table 8: Complete NAVS-G VNT results

A. LHSP	*Variance*	*SD*	*Estimate*	*SE*	*p*	*χ2*	**z**	*R^2*	*Power*
			patients with aphasia						
single predictor models									
**VAN**								0,193	
random effects									
item	1,254	1,12			0,0005	12,11			
participant	0,289	0,538			0,297	1,089			
fixed effects									
VAN					0,047	6,1058	6,106	0,1926	70%
2 vs. 1			-2,8577	1,2892	0,08		-2,217		
3 vs. 1			-2,1724	1,2661	0,129		-1,716		
3 vs. 2			0,6853	0,724	0,344		0,947		
**VAO**								0,054	
random effects									
item	1,521	1,233			0,000	14,426			
participant	0,293	0,541			0,289	1,123			
fixed effects									
VAO			1,0872	0,7375	0,14		1,474	0,0545	29%
**VT**								0,297	
random effects									
item	0,290	0,538			0,275	1,192			
participant	0,285	0,534			0,306	1,048			
fixed effects									
VT					0,002		17,46	0,2968	97%

ob2 vs. ob1			-2,822	1,1183	.02786		-2.524		
ob3 vs. ob1			-3,963	1,1933	.00416		-3.321		
op2 vs. ob1			-.7153	1,2905	.57856		-.554		
op3 vs. ob1			-1,8911	1,1551	.14323		-1.637		
ob3 vs. ob2			-1,1407	0,6956	.14323		-1.640		
op2 vs. ob2			2,1069	0,866	.02879		2.433		
op3 vs. ob2			0,9312	0,6453	.1828		1.443		
op2 vs. ob3			3,2477	0,9595	.00416		3.385		
op3 vs. ob3			2,072	0,7631	.02081		2.715		
op3 vs. op2			-1,1757	0,9134	.21795		-1.287		
**AATgs**								.059	
random effects									
item	1,094	1,046			0,062	3,471			
participant	0,069	0,263			0,841	0,040			
fixed effects									
AATgs			0,004	0,002	0,036		2,101		0%
**AATsy**								.048	
random effects									
item	2,221	1,490			0,000	21,808			
participant	0,000	0,000			0,998	0,000			
fixed effects									
AATsy			0,374	0,147	.011		2,555		64%
**frequency**								.157	
random effects									
item	0,860	0,928			0,016	5,752			
participant	0,281	0,530			0,304	1,058			
fixed effects									
frequency			-1,043	0,376	0,006		-2,774		82%
**Age**								.062	
random effects									
item	1,916	1,384			0,000	19,820			
participant	0,051	0,226			0,824	0,050			

fixed effects									
frequency			-0,072	0,029	.013		-2,478		63%
adjusted models									
**VAN + Age**								0,248	
random effects									
item	1,282	1,132			0,001	12,298			
participant	0,046	0,214			0,842	0,040			
fixed effects									
VAN					0,047	6,131		0,2481	74%
2 vs. 1			-2,888	1,299	0,078		-2,224		
3 vs. 1			-2,196	1,275	0,128		-1,722		
3 vs. 2			0,692	0,731	0,344		0,947		
age			-0,072	0,029	0,013		-2,483		
**VT + Age**								0,357	
random effects									
item	0,292	0,540			0,279	1,170			
participant	0,038	0,196			0,866	0,028			
fixed effects									
VT					0,001	17,579		0,3566	98%
ob2 vs.ob1			-2,845	1,121	0,028		-2,538		
ob3 vs.ob1			-4,001	1,197	0,004		-3,342		
op2 vs.ob1			-0,7184	1,2933	0,57856		-0,555		
op3 vs.ob1			-1,9032	1,158	0,14323		-1,644		
ob3 vs.ob2			-1,1558	0,6998	0,14323		-1,652		
op2 vs.ob2			2,127	0,869	0,029		2,447		
op3 vs.ob2			0,9421	0,6484	0,1828		1,453		
op2 vs.ob3			3,283	0,964	0,004		3,406		
op3 vs.ob3			2,098	0,767	0,021		2,735		
op3 vs.op2			-1,1848	0,9166	0,21795		-1,293		
age			-0,072	0,029	0,013		-2,493		
**VT + Age + frequency + AAT syntax**									

fixed effects									
VT					0,0238		11,26		
ob2 vs.ob1			-2,4321	1,110E+00	0,0949		-2,191		
ob3 vs.ob1			-2,6046	1,3939	0,1028		-1,869		
op2 vs.ob1			-0,2514	1,2962	0,8474		-0,194		
op3 vs.ob1			-1,1231	1,2005	0,4369		-0,936		
ob3 vs.ob2			-0,1724	0,8961	0,8474		-0,192		
op2 vs.ob2			2,1808	0,8444	0,0949		2,583		
op3 vs.ob2			1,309	0,6519	0,1028		2,008		
op2 vs.ob3			2,3532	1,0614	0,0949		2,217		
op3 vs.ob3			1,4815	0,7886	0,1028		1,879		
op3 vs.op2			-0,8717	0,8973	0,4369		-0,972		
frequency			-0,73591	0,43498	0,09068		-1,692		
age			-0,08327	0,03066	0,0066		-2,716		
AAT syntax			0,42061	0,15481	0,00659		2,717		
VAN + frequency + age, + AAT syntax									
fixed effects									
VAN					0,04722		1,679	0,3488	
2 vs. 1			-1,56801	1,4339	-1,094		0,411		
3 vs. 1			-1,49826	1,26803	-1,182		0,411		
3 vs. 2			0,06975	0,81548	0,086		0,932		
verb frequency			-0,87694	0,51064	0,08592		-1,717		
age			-0,08287	0,03074	0,00701		-2,696		
AAT syntax			0,42041	0,15497	0,00667		2,713		

Between population analysis

	Variance	*SD*	*Estimate*	*SE*	*p*	χ^2	*z*	*R-squared*	*Power**
single models									
Group								**0,206**	
random effects									
item	0,109	0,330			0,001	10,991			
participant	0,757	0,870			0,596	0,281			

fixed effects									
Group					0,000	29,329			100,00%
HS vs. RH*			0,416	0,623	0,504		0,668		
HS vs. LH*			2,478	0,470	0,000		5,275		
RH vs. LH*			2,062	0,510	0,000		4,046		
models with interactions									
**Group*VAN**								**0,238**	
random effects									
item	0,128	0,357			0,008	6,965			
participant	0,560	0,748			0,552	0,354			
fixed effects									
Group					0,000	32,816			
VAN					0,450	1,599			
Group*VAN					0,186	6,179			
**Group*VAO**								**0,270**	
random effects									
item	0,1169	0,3419			0,008	7,112			
participant	0,5706	0,7554			0,313	0,576			
fixed effects									
Group					0,000	34,184			
VAO					0,106	2,620			
Group*VAO					0,346	2,120			
**Group*VT**								**0,826**	
random effects									
item	0,13063	0,3614			0,924	0,009			
participant	0,01733	0,1316			0,569	0,325			
fixed effects									
Group					0,000	26,703			
VT					0,003	16,228			
Group*VT					0,226	10,596			
**Group*frequency**								**0,222**	
random effects									

item	0,1372	0,3705			0,094	2,797			
participant	0,3555	0,5963			0,533	0,389			
fixed effects									
Group					0,000	27,451			
frequency					0,047	3,951			
Group*frequency					0,021	7,718			
HS			0,372	0,464	0,423		0,802	0,031	11%
RH			-0,046	0,521	0,930		-0,087	0,000	3%
LH*			-1,043	0,376	0,006		-2,774	0,157	82%

**HP**	***Variance***	***SD***	***Estimate***	***SE***	***p***	***χ2***	**z**	***R²***	***Power***
single predictor models									
**VAN**								**0,008**	
random effects									
item	0,000	0,000			1,000	0,000			
participant	0,000	0,000			1,000	0,000			
fixed effects									
VAN					0,128	0,938			7%
**VAO**								0,003	
random effects									
item	0,000	0,000			1,000	0,000			
participant	0,000	0,000			1,000	0,000			
fixed effects									
VAO			-0,184	0,821	0,822		-0,224		8%
**VT**								**0,038**	
random effects									
item	0,000	0,000			1,000	0,000			
participant	0,000	0,000			1,000	0,000			
fixed effects									
VT					0,921	0,927	0,8932		3%

ob2 vs. ob1	-2,316E-13	1,419	1	0
ob3 vs. ob1	-0,9275	1,423	0,949	-0,652
op2 vs. ob1	-0,7006	1,231	0,949	-0,569
op3 vs. ob1	-2,152E-13	1,419	1	0
ob3 vs. ob2	-0,9275	1,423	0,949	-0,652
op2 vs. ob2	-0,7006	1,231	0,949	-0,569
op3 vs. ob2	1,641E-14	1,419	1	0
op2 vs. ob3	0,2269	1,235	1	0,184
op3 vs. ob3	0,9275	1,423	0,949	0,652
op3 vs. op2	0,7006	1,231	0,949	0,569

B. RHSP	*Variance*	*SD*	*Estimate*	*SE*	*p*	*χ2*	z	*R^2*	*Power*
**single predictor models**									
**VAN**								**0,103**	
random effects									
item	0,000	0,000			1,000	0,000			
participant	0,000	0,000			1,000	0,000			
fixed effects									
VAN					0,517	1,322			26%
**VAO**								**0,101**	
random effects									
item	0,000	0,000			1,000	0,000			
participant	0,000	0,000			1,000	0,000			
fixed effects									
VAO			1,220	1,124	0,278		1,086		24%
VT - the estimates of this model are not reliable									

C. healthy participants

	Variance	*SD*	*Estimate*	*SE*	*p*	*χ2*	z	*R^2*	*Power*
single predictor models									

**VAN**								0,008	
random effects									
item	0,000	0,000			1,000	0,000			
participant	0,000	0,000			1,000	0,000			
fixed effects									
VAN					0,128	0,938			7%
**VAO**								0,003	
single predictor models									
random effects									
item	0,000	0,000			1,000	0,000			
participant	0,000	0,000			1,000	0,000			
fixed effects									
VAO			-0,184	0,821	0,822		-0,224		8%
**VT**								0,038	
random effects									
item	0,000	0,000			1,000	0,000			
participant	0,000	0,000			1,000	0,000			
fixed effects									
VT					0,921	0,927			3%

Supplementary Table 9: Complete NAVS-G ASPT results

LHSP	*Variance*	*SD*	*Estimate*	*SE*	*p*	*χ2*	z	*R^2*	*Power*
single predictor models									
**VAN**								**0,039**	
random effects									
item	0,146	0,382			0,765	0,089			
participant	31,202	5,586			0,000	103,360			

fixed effects									
VAN					0,000		15,919	0,039035	0,66
2 vs. 1			-3,5965	1,2248	0,00996		-2,936		
3 vs. 1			-2,2592	1,1763	0,05478		-1,921		
3 vs. 2			1,3373	0,5314	0,01777		2,517		
VAO								0,006	
random effects									
item	0,947	0,973			0,109	2,576			
participant	27,614	5,255			0,000	99,941			
fixed effects									
VAO			-0,882	0,637	0,166		-1,386		24%
**VT**								**0,039**	
random effects									
item	0,076	0,276			0,875	0,025			
participant	31,973	5,655			0,000	103,790			
fixed effects									
VT					0,002	16,896			78%
**NIHSS**								**0,241**	
random effects									
item	0,994	0,997			0,084	2,976			
participant	7,340	2,709			0,000	25,374			
fixed effects									
NIHSS			-0,924	0,418	0,027		-2,209		61%
**mRS**								**0,280**	
random effects									
item	1,013	1,007			0,082	3,031			
participant	6,703	2,589			0,000	26,090			
fixed effects									
mRS			-2,906	1,212	0,016		-2,938		69%
**BI**								**0,281**	
random effects									
item	1,060	1,029			0,073	3,207			

participant	4,466	2,113			0,000	12,939			
fixed effects									
BI*			0,170	0,063	0,006		2,721		84%
adjusted models									
**VAN + NIHSS**								0,327	
random effects									
item	0,128	0,358			0,792	0,069			
participant	7,612	2,759			0,000	26,176			
fixed effects									
VAN					0,000	16,026			87%
2 vs. 1			-3,586	1,220	0,010		-2,939		
3 vs. 1			-2,261	1,174	0,054		-1,925		
3 vs. 2			1,325	0,525	0,017		2,523		
NIHSS			-0,952	0,431	0,027		-2,211		
**VT + NIHSS**								0,329	
random effects									
item	0,056	0,238			0,907	0,014			
participant	7,684	2,772			0,000	26,365			
fixed effects									
VT					0,002		16,896	0,32872	87%
ob2ob1			-2,24222	1,26263	0,1082		-1,776		
ob3 vs. ob1			-4,205	1,402	0,024		-2,998		
op2 vs. ob1			-2,24243	1,188326	0,0986		-1,887		
op3 vs. ob1			-3,416	1,210	0,024		-2,823		
ob3 vs. ob2			-1,96233	0,993949	0,0967		-1,974		
op2 vs. ob2			-0,00021	0,741602	0,9998		0		
op3 vs. ob2			-1,17369	0,724765	0,1317		-1,619		
op2 vs. ob3			1,962122	0,896349	0,0953		2,189		
op3 vs. ob3			0,788637	0,829337	0,3796		0,951		
op3 vs. op2			-1,17349	0,584159	0,0967		-2,009		
NIHSS			-0,9556	0,4338	0,02760		-2,203		
VT + Barthel									

fixed effects									
VT					0,00208		16,835	0,389095	0,89
ob2 vs. ob1			-2,27673	1,276271	0,10640	-1,784			
ob3 vs. ob1			-4,25276	1,415509	0,02310	-3,004			
op2 vs. ob1			-2,27723	1,200333	0,09640	-1,897			
op3 vs. ob1			-3,46884	1,224748	0,02310	-2,832			
ob3 vs. ob2			-1,97603	1,004822	0,09640	-1,967			
op2 vs. ob2			-0,0005	0,755128	0,99950	-0,001			
op3 vs. ob2			-1,19211	0,738454	0,13310	-1,614			
op2 vs. ob3			1,975536	0,904304	0,09640	2,185			
op3 vs. ob3			0,783919	0,834165	0,38590	0,94			
op3 vs. op2			-1,19162	0,595039	0,09640	-2,003			
Barthel			0,17321	0,06342	0,00631	2,731			
**between population analyses**									
	Variance	***SD***	***Estimate***	***SE***	***p***	**χ^2**	***z***	***R-squared***	***Power***
Single models									
**Group**								**0,093**	
random effects									
item	0,451	0,672			0,128	2,321			
participant	4,350	2,086			0,000	89,156			
fixed effects									
Group					0,107	4,464			36%
**Models with interactions**									
**Group*VAN**								**0,102**	
random effects									
item	0,213	0,462			0,475	0,512			
participant	4,815	2,194			0,000	93,049			
fixed effects									
Group					0,093	4,748			
VAN					0,074	5,201			
Group*VAN					0,175	6,338			

**Group*VAO**								**0,113**	
random effects									
item	0,440	0,663			0,129	2,300			
participant	4,457	2,111			0,000	89,946			
fixed effects									
Group					0,095	4,698			
VAO					0,647	0,209			
Group*VAO					0,127	4,122			
**Group*VT**								**0,124**	
random effects									
item	0,066	0,258			0,803	0,062			
participant	4,771	2,184			0,000	93,044			
fixed effects									
Group					0,105	4,509			
VT					0,073	8,574			
Group*VT					0,530	7,064			

Supplementary Table 10: Complete NAVS-G SPPT results

A. NAVS-G SPPT in LHSP									
	Variance	***SD***	***Estimate***	***SE***	***p***	***χ2***	**z**	***R^2***	***Power***
**Single predictor models**									
**Canonicity**								**0,129**	
random effects									
item	2,459	1,568			0,000	55,380			
participant	3,318	1,821			0,000	118,240			
fixed effects									

Canonicity			2,318	0,661	0,000		3,504	0,1292566	98%
**Canonicity without relatives**								**0,108**	
random effects									
item	0,321	0,567			0,317	1,003			
participant	10,601	3,256			0,000	88,993			
fixed effects									
Canonicity without relatives			2,618	0,551	<.001		4,748	0,1079276	21%
Sentence type								0,394	
random effects									
item	0,000	0,000			1,000	0,000			
participant	3,478	1,865			0,000	122,960			
fixed effects									
**Sentence type**					**<.001**	**82,395**			**100%**
P vs. A			-3,981	0,8486	0,00000543		-4,691		
OW vs. SW			-1,3988	0,4998	0,0057		-2,799		
OR vs. SR*			-2,0581	0,4672	0,0000151		-4,405		
SR vs. A			-4,7911	0,8618	6,76E-08		-5,56		
SW vs. A			-2,4767	0,8461	0,00427		-2,927		
OR vs. P			-2,8681	0,5018	3,64E-08		-5,716		
OW vs. P			0,1055	0,4551	0,81664		0,232		
OR vs. OW			-2,9736	0,5067	2,19E-08		-5,869		
SR vs. SW			-2,3144	0,5152	0,0000117		-4,492		
OR vs. non-OR			-12,2722	1,6902	3,85E-12		-7,261		
**NIHSS**								**0,090**	
random effects									
item	3,796	1,948			0,000	88,997			
participant	2,394	1,547			0,000	83,745			
fixed effects									
NIHSS			-0,466	0,205	0,023		-2,277		66%
**AAT.ss**								**0,177**	
random effects									
item	3,785	1,946			0,000	89,088			

participant	1,470	1,213			0,000	47,805			
fixed effects									
AAT.ss			0,320	0,083	0,000		3,867		96%
**AAT.sy***								**0,135**	
random effects									
item	3,790	1,947			0,000	88,984			
participant	1,934	1,391			0,000	67,145			
fixed effects									
AAT.sy			0,845	0,278	0,002		3,038		85%
Aph.severity								0,168	
random effects									
item	3,774	1,943			0,000	72,953			
participant	1,665	1,290			0,000	49,338			
fixed effects									
Aph.se			1,125	0,337	0,001		3,338		0%
**Token Test**								**0,111**	
random effects									
item	3,930	1,982			0,000	67,291			
participant	2,537	1,593			0,000	70,892			
fixed effects									
Token Test			0,178	0,080	0,025		2,236		0%
**adjusted models**									
Canonicity + AAT.ss								0,307	
random effects									
item	2,443	1,563			0,000	55,394			
participant	1,457	1,207			0,000	47,721			
fixed effects									
Canonicity			2,318	0,661	0,000		3,506		96%
AAT.ss			0,320	0,083	0,000		3,872		
**Canonicity without relatives + AAT.ss**								0,449	

random effects									
item	0,3365	0,5801			0,300	1,073			
participant	4,2219	2,0547			0,000	35,170			
fixed effects									
canonicity without relatives			2,6481	0,5583	0,000		4,743	0,4446722	99%
AAT.ss			0,5021	0,164	0,002		3,062		
**Sentence type + AAT.ss***								**0,569**	
random effects									
item	0,000	0,000			1,000	0,000			
participant	1,524	1,235			0,000	49,173			
fixed effects									
Sentence type					0,000	82,377		0,5689824	100%
P vs. A*			-3,993	0,848	0,000		-4,711		
OW vs. SW*			-1,407	0,502	0,006		-2,805		
OR vs. SR*			-2,044	0,466	0,000		-4,390		
SR vs. A*			-4,807	0,861	0,000		-5,583		
SW vs. A*			-2,480	0,845	0,004		-2,935		
OR vs. P*			-2,858	0,501	0,000		-5,701		
OW vs. P			0,1062	0,4575	0,8164		0,232		
OR vs. OW			-2,9645	0,5064	0,0000		-5,854		
SR vs. SW*			-2,327	0,517	0,000		-4,500		
non-OR vs. OR*			-12,239	1,685	0,000		-7,264		
relative vs. non-relative			-3,0568	0,5889	0,00000		-5,191		
AAT.ss			0,329	0,085	0,000		3,866		
**Canonicity + AAT syntax**									
fixed effects									
Canonicity			-2,3161	0,6608	0,000457		-3,505		
AAT syntax			0,8436	0,2773	0,002348		3,042		
Canonicity without relatives + AAT syntax									
fixed effects									

Canonicity			2,6405	0,5578	0,00000		4,734		
AAT syntax			1,6817	0,5683	0,00309		2,959		
**sentence type + AAT syntax**									
fixed effects									
sentence type					0,00000	69,247			
P vs. A			-3,9791	0,8471	0,0000	-4,698			
OW vs. SW			-1,3985	0,5000	0,0057	-2,797			
OR vs. SR			-2,0639	0,4685	0,0000	-4,405			
SR vs. A			-4,7885	0,8603	0,0000	-5,566			
SW vs. A			-2,4753	0,8447	0,0042	-2,93			
OR vs. P			-2,8732	0,5031	0,0000	-5,712			
OW vs. P			0,1054	0,4554	0,8170	0,231			
OR vs. OW			-2,9786	0,5079	0,0000	-5,864			
SR vs. SW			-2,3132	0,5154	0,0000	-4,488			
OR vs. non-OR			-12,2928	1,6960	0,0000	-7,248			
relative vs. non-relative			-3,0543	0,5885	0,0000	-5,190			
AAT syntax			0,8547	0,2834	0,00256	3,016			
**Canonicity + NIHSS**								**0,219**	
random effects									
item	2,450	1,565			0,000	55,355			
participant	2,373	1,541			0,000	83,690			
fixed effects									
Canonicity			2,316	0,661	0,000		3,505		96%
NIHSS			-0,465	0,204	0,023		-2,281		
**Canonicity without relatives + NIHSS**								**0,274**	
random effects									
item	0,330	0,574			0,307	1,044			
participant	8,043	2,836			0,000	68,434			
fixed effects									
Canonicity			2,629	0,554	0,000		4,742		100%
NIHSS			-0,783	0,420	0,062		-1,864		

**Sentence type + NIHSS**								**0,482**	
random effects									
item	0,000	0,000			1,000	0,000			
participant	2,488	1,577			0,000	86,779			
fixed effects									
Sentence type					<.001	69,185			100%
P vs. A*			-3,974	0,845	0,000		-4,702		
OW vs. SW*			-1,401	0,500	0,006		-2,800		
OR vs. SR*			-2,056	0,467	0,000		-4,403		
SR vs. A*			-4,785	0,858	0,000		-5,574		
NIHSS			-0,477	0,210	0,023		-2,276		
**Sentence type + education**									
fixed effects									
Sentence type					1,54E-13		69,155	0,3944695	100%
**between population analysis**									
	Variance	***SD***	***Estimate***	***SE***	***p***	***χ2***	***z***	***R-squared***	***Power***
Single predictor models									
**Group**								**0,229**	
random effects									
item	2,452	1,566			0,000	186,180			
participant	2,896	1,702			0,000	149,120			
fixed effects									
Group					0,00000	35,736			100%
HS vs. RH			1,078	0,625	0,084		1,727		
HS vs. LH			3,857	0,620	0,000		6,226		
RH vs. LH			2,779	0,650	0,000		4,278		
models with interactions									
**Group*canonicity**								**0,317**	
random effects									

item	2,472	1,572			0,000	120,900			
participant	1,920	1,386			0,000	149,770			
fixed effects									
Group					0,000	40,021			
Canonicity					0,000	12,526			
Group*Canonicity					0,512	0,774			
**Group*canonicity without relatives**								**0,143**	
random effects									
item	3,800	1,949			0,075	3,170			
participant	0,316	0,562			0,000	80,989			
fixed effects									
Group					0,000	15,269			
Canonicity without relatives					0,000	17,310			
Group*Canonicity without relatives					0,029	7,071			
Canonical									*56%*
HS vs. RH			0,641	1,036	0,536		0,619		
HS vs. LH			2,450	0,901	0,020		2,718		
RH vs. LH			1,809	0,950	0,085		1,904		
Non-Canonical									97%
HS vs. RH			2,426	1,104	0,028		2,197		
HS vs. LH			4,705	1,086	0,000		4,333		
RH vs. LH			2,280	0,986	0,028		2,312		
**Group*Sentence type**								0,441	
random effects									
item	2,723	1,650			0,068	3,343			
participant	0,128	0,358			0,000	155,900			
fixed effects									
Group					0,000	32,619			
Sentence type					0,000	101,713			
Group*Sentence type					0,013	22,341			

Active									
Group					0,973	0,056		0,002	1%
Passive									
Group					0,198	3,242		0,182	9%
Subject Wh-questions									
Group					0,051	5,934		0,179	43%
HS>RH			0,621	1,303	0,634		0,477		
HS>LH			2,955	1,128	0,026		2,621		
RH>LH			2,334	1,187	0,074		1,967		
Object Wh-questions									
Group					0,013	8,691		0,272	66%
HS>RH			3,010	1,406	0,048		2,141		
HS>LH			4,905	1,479	0,003		3,316		
RH>LH			1,895	1,333	0,155		1,421		
Subject-Relative									
Group					0,000	24,200		0,349	94%
HS>RH			0,509	1,309	0,698		0,389		
HS>LH			5,489	1,486	0,001		3,693		
RH>LH			4,980	1,631	0,003		3,054		
Object-Relative									
Group					0,000	30,263		0,294	100%
HS>RH			0,566	0,689	0,411		0,822		
HS>LH			3,771	0,794	0,000		4,749		
RH>LH			3,205	0,799	0,000		4,011		
Group*Sentence type*Age								0,655	
random effects									
item	2,179	1,476			0,053	3,754			
participant	0,143	0,379			0,000	129,550			
fixed effects									
Group					0,000	38,928			
Sentence type					0,000	83,104			

Age			-0,066	0,024	0,006		-2,728		
*Group*Age:*					.032	12.238			
effect of age in HP			-0,09533	0,02877	0,002763		-3,314		98%
effect of age in RH			-0,06288	0,02883	0,0438		-2,181		62,50%
effect of age in LH			-0,06899	0,05757	0,2308		-1,198		28,50%
*Sentence Type*Age*					0,032	12,238			
Active					0,9576	0,3782			
Passive					0,9576	0,6301			
Subject Wh-questions					0,9576	0,0865			
Object Wh-questions					0,9576	3,3847			
Subject Relative					0,9576	1,1319			
Object-Relative					0,9576	1,8153			
*Group*Sentence Type*Age*					0,021835	23,7732		.654	
sentence type*age in HS					0,14148	9,3946		.243	
sentence type*age in RH					0,09609	12,209		.103	
sentence type*age in LH					0,5175	4,2252		.031	
**Group*Sentence type*Education**								.947	
random effects									
item	1,490	1,221			0,046	3,974			
participant	0,150	0,387			0,000	97,701			
fixed effects									
Group					0,000	47,131			
Sentence type					0,000	75,390			
Ed.Qual			1,423	0,388	0,000		3,671		
*Sentence Type*Ed.q.*					0,067	10,300			
*Group*Sentence Type*Ed.q.*					0,0002486	36,714			
Active					0,79725	1,264			
Passive					0,79725	1,3334			
Subject Wh-questions					0,8648	0,2906			
Object Wh-questions					0,5538	3,3789			
Subject Relative					0,8648	0,4065			

Object-Relative	.341 (0,5*)	5,732			
HS	0.000	4,667		.49	100%
RH	0,0303	2,323		.24	55%
LH	0,1255	1,532		.007	33,50%

*uncorrect

**B**	**NAVS-G SPPT in RHSP without aphasia**								
	Variance	***SD***	***Estimate***	***SE***	***p***	***χ2***	**z**	***R^2***	***Power***
**Single predictor models**									
**Canonicity**								**0,073**	
random effects									
item	2,284	1,511			0,000	28,700			
participant	0,958	0,979			0,008	6,971			
fixed effects									
Canonicity			1,438	0,780	0,065		1,844	0,07347395	49%
**Canonicity without relatives**								**0,040**	
random effects									
item	2,460	1,568			0,000	13,149			
participant	0,457	0,676			0,455	0,559			
fixed effects									
Canonicity without relatives			1,017	1,086	0,349		0,936		19%
**Sentence type**								**0,234**	
random effects									
item	1,370	1,171			0,000	15,134			
participant	0,989	0,994			0,008	7,075			
fixed effects									
Sentence type					0,018	13,672			82%

P vs. A			0	1,672	1		0		
OW vs. SW			-1,584	1,178	0,3248		-1,344		
OR vs. SR*			-1,93	0,9999	0,1787		-1,93		
SR vs. A			-1,674	1,423	0,3419		-1,177		
SW vs. A			-0,779	1,511	0,6735		-0,516		
OR vs. P			-3,604	1,359	0,0401		-2,652		
OW vs. P			-2,363	1,382	0,2182		-1,71		
OR vs. OW			-1,241	0,9574	0,3248		-1,296		
SR vs. SW			-0,895	1,222	0,5798		-0,733		
OR vs. non-OR			-9,599	3,176	0,025		-3,023		
**Ed. Qual.**								**0,162**	
random effects									
item	2,980	1,726			0,000	40,203			
participant	0,276	0,525			0,285	1,142			
fixed effects									
Ed.qual			1,568	0,567	0,006		2,768		80%
**Ed. Grad.**								**0,039**	
random effects									
item	3,050	1,747			0,000	40,733			
participant	0,835	0,914			0,016	5,752			
fixed effects									
Ed.grad			0,939	0,613	0,126		1,531		39%
**Age**								**0,103**	
random effects									
item	3,073	1,753			0,000	40,929			
participant	0,650	0,806			0,042	4,141			
fixed effects									
Age			-0,063	0,029	0,029		-2,181		70%
**adjusted models**									
**Canonicity + Ed.qual**								**0,234**	

random effects									
item	2,258	1,503			0,000	28,467			
participant	0,269	0,518			0,292	1,109			
fixed effects									
Canonicity			1,432	0,775	0,065		1,847		43%
Ed.qual			1,562	0,564	0,006		2,767		
**Canonicity without relatives + Ed.qual**								**0,140**	
random effects									
item	2,461	1,569			0,000	13,207			
participant	0,098	0,313			0,030	0,863			
fixed effects									
Canonicity without relatives			1,027	1,091	0,346		0,942		16%
Ed.qual			1,150	0,718	0,109		1,602		
**Sentence type + Ed.qual**								0,376	
random effects									
item	1,357	1,165			0,000	14,596			
participant	0,278	0,528			0,287	1,133			
fixed effects									
Sentence type					0,018	13,671			92%
OR vs. SR*			-1,930	1,000	0,179		-1,930		
OR vs. P*			-3,604	1,359	0,040		-2,652		
OR vs. OW*			-1,241	0,957	0,325		-1,296		
non-OR vs. OR*			-9,599	3,176	0,025		-3,023		
Non-Relvs.Rel*			-1,919	0,826	0,020		-3,250		
Ed.qual			1,566	0,566	0,006		2,769		

**C**	**NAVS-G SPPT in HP**								
	Variance	***SD***	***Estimate***	***SE***	***p***	***χ2***	**z**	***R^2***	***Power***
**Single predictor models**									

**Canonicity**								**0,119**	
random effects									
item	3,506	1,872			0,000	31,679			
participant	3,913	1,978			0,000	32,56			
fixed effects									
Canonicity			1,8503	0,9296	0,0465		1,99	0,11891541	56%
**Canonicity without relatives**								**0,000**	
random effects									
item	1,308	1,144			1,000	0,000			
participant	0,000	0,000			0,437	0,604			
fixed effects									
Canonicity without relatives			0,003	0,826	0,997		0,003	0,000000	6%
**Sentence type**								**0,239**	
random effects									
item	0,134	0,366			0,626	0,238			
participant	3,743	1,935			0,000	31,250			
fixed effects									
Sentence type					0,000	42,442			100%
P vs. A			0	1,672	1		0		
OW vs. SW			-1,584	1,178	0,3248		-1,344		
OR vs. SR*			-1,93	0,9999	0,1787		-1,93		
SR vs. A			-1,674	1,423	0,3419		-1,177		
SW vs. A			-0,779	1,511	0,6735		-0,516		
OR vs. P			-3,604	1,359	0,0401		-2,652		
OW vs. P			-2,363	1,382	0,2182		-1,71		
OR vs. OW			-1,241	0,9574	0,3248		-1,296		
SR vs. SW			-0,895	1,222	0,5798		-0,733		
OR vs. non-OR			-9,599	3,176	0,025		-3,023		
**Ed. Qual.**								**0,188**	
random effects									
item	4,345	2,084			0,000	43,723			

participant	0,307	0,554			0,254	1,302			
fixed effects									
Ed.qual			1,441	0,266	0,000		5,427		100%
**Ed. Grad.**								**0,114**	
random effects									
item	4,552	2,134			0,000	44,785			
participant	1,869	1,367			0,000	16,684			
fixed effects									
Ed.grad			1,488	0,468	0,001		3,181		89%
**Age**								**0,243**	
random effects									
item	4,733	2,176			0,000	45,582			
participant	1,856	1,362			0,000	14,912			
fixed effects									
Age			-0,095	0,029	0,001		-3,314		96%

**adjusted models**									
**Canonicity + Ed.qual**								0,28	
random effects									
item	3,219	1,794			0,000	30,430			
participant	0,300	0,547			0,264	1,254			
fixed effects									
Canonicity			1,790	0,902	0,047		1,985		53%
Ed.qual			1,437	0,265	0,000		5,429		
**Canonicity without relatives + Ed.qual**								0,059	
random effects									
item	0,707	0,841			1,000	0,000			
participant	0,000	0,000			0,684	0,165			
fixed effects									

	Variance	SD	Estimate	SE	p	χ2	z	R^2	Power
Canonicity without relatives			-0,005	0,826	0,995		-0,006		7%
Ed.qual			0,533	0,447	0,233		1,193		
**Sentence type + Ed.qual**								0,507	
random effects									
item	0,135	0,368			0,594	0,284			
participant	0,275	0,524			0,308	1,041			
fixed effects									
Sentence type					0,000	42,457			100%
OR vs. SR*			-2,665	0,677	0,000		-3,935		
OR vs. P*			-4,113	1,106	0,000		-3,720		
OR vs. OW*			-3,381	0,845	0,000		-4,001		
non-OR vs. OR*			-13,485	2,215	0,000		-6,087		
Non-Relvs.Rel*			-2,898	0,730	0,000		-3,969		
Ed.qual			1,426	0,263	0,000		5,417		

Supplementary Table 11: Complete NAVS-G SCT results

**NAVS-G SCT in patients with aphasia**									
	Variance	***SD***	***Estimate***	***SE***	***p***	***χ2***	**z**	***R^2***	***Power***
**single predictor models**									
**Canonicity**								0,088	
random effects									
item	0,424	0,651			0,066	3,390			
participant	0,879	0,937			0,002	9,282			
fixed effects									
Canonicity			1,329	0,466	0,004		2,851		83%
**Canonicity without relatives**								0,092	
random effects									

item	0,000	0,000			0,999	0,000			
participant	1,747	1,322			0,008	6,936			
fixed effects									
Canonicity without relatives			1,424	0,599	0,018		2,376		69%
**Sentence type***								0,19	
random effects									
item	0,126	0,355			0,499	0,457			
participant	0,894	0,945			0,002	9,299			
fixed effects									
Sentence type					0,004	17,211			100%
**NIHSS**								0,058	
random effects									
item	0,796	0,892			0,003	8,603			
participant	0,500	0,707			0,050	3,827			
fixed effects									
NIHSS			-0,254	0,121	0,036		-2,098		67%
**BI**								0,047	
random effects									
item	0,799	0,894			0,003	8,638			
participant	0,519	0,720			0,050	3,842			
fixed effects									
BI			0,044	0,022	0,042		2,032		58%
**AAT.ss***								0,096	
random effects									
item	0,798	0,893			0,003	8,614			
participant	0,283	0,532			0,240	1,379			
fixed effects									
AAT.ss			0,161	0,053	0,003		3,005		91%
**AAT.sy**								0,055	

random effects									
item	0,793	0,891			0,003	8,570			
participant	0,523	0,723			0,040	4,228			
fixed effects									
AAT.sy			0,368	0,187	0,050		1,961		53%
**Aph.se**								0,108	
random effects									
item	1,803	1,343			0,000	15,917			
participant	0,599	0,774			0,073	3,213			
fixed effects									
Aph.se			0,701	0,270	0,009		2,599		0%
**adjusted models**									
**Canonicity + AAT.ss**								0,185	
random effects									
item	0,424	0,651							
participant	0,281	0,530							
fixed effects									
Canonicity			1,332	0,467	0,004		2,853		85%
AAT.ss			0,161	0,053	0,003		3,015		
**Canonicity without relatives + AAT.ss**								0,286	
random effects									
item	0,000	0,000		1,000	0,000				
participant	0,409	0,640		0,488	0,481				
fixed effects									
Canonicity			1,435	0,602	0,017		2,385		72%
AAT.ss			0,232	0,082	0,005		2,821		
**Sentence type + AAT.ss**								0,287	
random effects									

item	0,125	0,353							
participant	0,280	0,529							
fixed effects									
Sentence type*					0,004	17,574			97%
ORvs.non-OR			-5,812	1,700	0,006		-3,418		
Relvs.non-Rel			-1,145	0,454	0,012		-2,519		
AAT.ss			0,162	0,053	0,002		3,026		

**NAVS-G SCT in RHSP**									
	Variance	***SD***	***Estimate***	***SE***	***p***	***χ^2***	***z***	***R-squared***	***Power****
**single predictor models**									
**Canonicity**								0,004	
random effects									
item	0,000	0,000			1,000	0,000			
participant	17,490	4,182			0,001	11,054			
fixed effects									
Canonicity			0,576	0,774	0,457		0,744		14%
**Canonicity without relatives**								0,000	
random effects									
item	0,361	0,601			0,853	0,034			
participant	25,314	5,031			0,012	6,369			
fixed effects									
Canonicity without relatives			0,005	1,136	0,996		0,004		6%
**Sentence type**								0,011	
random effects									
item	0,000	0,000			1,000	0,000			
participant	18,540	4,306			0,001	11,286			
fixed effects									
Sentence type					0,6982	0,1503			18%

c. Healthy participants

	Variance	SD	Estimate	SE	p	χ²	z	R-squared	Power*
**single predictor models**									
**Canonicity**								0,002	
random effects									
item	0,233	0,483			0,864	0,029			
participant	8,539	2,922			0,002	9,817			
fixed effects									
Canonicity			0,318	0,823	0,699		0,386		8%
**Canonicity without relatives**								0,000	
random effects									
item	7,335	2,708			0,683	0,167			
participant	0,904	0,951			0,092	2,843			
fixed effects									
Canonicity without relatives			0,000	1,148	1,000		0,000		12%
**Sentence type**								0,007	
random effects									
item	0,141	0,376			0,918	0,011			
participant	9,114	3,019			0,002	9,846			
fixed effects									
Sentence type					0,9819	0,7189			8%

Supplementary Table 12: Complete NAT-G results

NAT-G LHSP

	Variance	SD	Estimate	SE	p	χ2	z	R^2	Power
single predictor models									
**Canonicity**								**0,066**	

random effects									
item	1,534	1,238			0,001	10,678			
participant	12,847	3,584			0,000	186,760			
fixed effects									
Canonicity			2,228	0,652	0,001		3,416		96%
**Canonicity without relatives**								**0,078**	
random effects									
item	0,000	0,000			1,000	0,000			
participant	21,440	4,630			0,000	90,186			
fixed effects									
Canonicity without relatives			2,883	0,652	0,000		4,425		91%
**Sentence type**								**0,194**	
random effects									
item	0,000	0,000			1,000	0,000			
participant	14,650	3,827			0,000	202,910			
fixed effects									
Sentence type					0,000	39,635			99%
**NIHSS**								**0,235**	
random effects									
item	2,480	1,575			0,000	21,597			
participant	9,558	3,092			0,000	143,150			
fixed effects									
NIHSS			-1,042	0,468	0,026		-2,227		77%
**AAT.sy***								**0,306**	
random effects									
item	2,451	1,566			0,000	21,470			
participant	6,171	2,484			0,000	89,345			
fixed effects									
AAT.sy			1,630	0,551	0,003		2,958		89%
**Aph.se**								**0,287**	
random effects									
item	2,462	1,569			0,000	21,443			

participant	5,531	2,352			0,000	90,100			
fixed effects									
Aph.se			1,805	0,654	0,006		2,761		0%
**Token Test**								**0,259**	
random effects									
item	2,473	1,573			0,000	21,418			
participant	5,333	2,309			0,000	89,142			
fixed effects									
Token Test			0,319	0,126	0,011		2,530		0%
**AAT.ss***								**0,41**	
random effects									
item	2,496	1,580			0,000	21,831			
participant	3,889	1,972			0,000	58,158			
fixed effects									
AAT.ss*			0,613	0,157	0,000		3,916		99%
**adjusted models**									
**Canonicity + AAT.ss**								**0,484**	
random effects									
item	1,599	1,264			0,001	11,146			
participant	4,077	2,019			0,000	59,749			
fixed effects									
Canonicity			2,273	0,663	0,001		3,426		99%
AAT.ss			0,630	0,161	0,000		3,921		
**Canonicity without relatives + AAT.ss**								0,567	
random effects									
item	0,000	0,000			1,000	0,000			
participant	3,662	1,914			0,000	26,416			
fixed effects									
Canonicity without relatives			2,861	0,637	0,000		4,489		100%
AAT.ss			0,627	0,182	0,001		3,444		
**Sentence type + AAT.ss**								0,615	

	Variance	SD	Estimate	SE	p	χ2	z	R^2	Power
random effects									
item	0,000	0,000			1,000	0,000			
participant	4,654	2,157			0,000	65,341			
fixed effects									
Sentence type					0,000	40,124			100%
Pvs.A			-4,137	1,035	0,000		-3,996		
OWvs.SW			-2,258	0,780	0,005		-2,895		
SRvs.A			-5,067	1,073	0,000		-4,722		
ORvs.P			-2,169	0,730	0,005		-2,972		
ORvs.OW			-2,403	0,743	0,002		-3,236		
SRvs.SW			-3,421	0,832	0,000		-4,113		
ORvs.non-OR			-10,472	2,435	0,000		-4,301		
Rel vs. Non-Rel			-3,104	0,712	0,000		-4,261		
AAT.ss			0,679	0,171	0,000		3,963		

**between group analyses**									
	Variance	***SD***	***Estimate***	***SE***	***p***	***χ2***	**z**	***R^2***	***Power***
**single predictor models**									
Group								0,196	
random effects									
item	5,429	2,330			0,000	22,629			
participant	1,239	1,113			0,000	179,400			
fixed effects									
Group					0,001	13,309			88,00%
HSvs.RH			-1,431	1,024	0,162		1,398		
HSvs.LH			-3,787	0,981	0,000		3,861		
RHvs.LH			-2,355	1,041	0,036		2,262		
**models with interactions**									
**Group*Canonicity**								**0,199**	
random effects									
item	0,795	0,892			0,001	11,919			

participant	5,831	2,415			0,000	184,010		
fixed effects								
Group					0,002	12,902		
Canonicity					0,001	10,756		
Group*Canonicity					0,150	3,792		
Group*Canonicity without relatives							0,120	
random effects								
item	0,000	0,000			1,000	0,000		
participant	7,140	2,672			0,000	82,725		
fixed effects								
Group					0,067	5,396		
Canonicity without relatives					0,001	11,093		
Group*Canonicity without relatives					0,018	8,031		
Canonical								
Group					0,346	2,122	0,065	3,00%
HSvs.RH			0,719	1,573	0,648	0,457		
HSvs.LH			2,767	1,632	0,270	1,696		
RHvs.LH			2,048	1,722	0,351	1,190		
Non-Canonical								
Group					0,002	12,692	0,249	77,00%
HSvs.RH			1,925	1,287	0,135	1,496		
HSvs.LH			4,834	1,281	0,000	3,774		
RHvs.LH			2,908	1,293	0,037	2,250		
**Group*Sentence type**							**0,204**	
random effects								
item	7,216	2,686			0,835	0,044		
participant	0,027	0,165			0,000	198,330		
fixed effects								
Group					0,010	9,237		
Sentence type					0,000	30,745		
Group*Sentence type					0,059	17,763		
Active								
Group					0,548	1,205	0,081	13%

Passive				
Group	0,642	0,887	0,014	0%
Subject Wh-Questions				
Group	0,736	0,613	0,014	0%
Object Wh-Questions				
Group	0,214	3,085	0,134	7%
Subject Relative				
Group	0,680	0,772	0,009	1%
Object Relative				
Group	0,120	4,242	0,189	13%

NAT-G RHSP

	Variance	*SD*	*Estimate*	*SE*	*p*	*χ2*	*z*	R^2	*Power*
**single predictor models**									
**Canonicity**								**0,046**	
random effects									
item	2,053	1,433			0,037	4,354			
participant	1,946	1,395			0,018	5,633			
fixed effects									
Canonicity			1,185	0,944	0,210		1,254		17%
**Canonicity without relatives**								**0,000**	
random effects									
item	0,000	0,000			1,000	0,000			
participant	0,000	0,000			1,000	0,000			
fixed effects									
Canonicity without relatives			0,000	1,007	1,000		0,000		5%
**Sentence type**								**0,106**	
random effects									

item	0,775	0,880			0,321	0,984			
participant	2,005	1,416			0,020	5,394			
fixed effects									
Sentence type					0,262	6,483			48%
